# Survival trends in critically ill HIV-infected patients in the highly active antiretroviral therapy era

**DOI:** 10.1186/cc9056

**Published:** 2010-06-09

**Authors:** Isaline Coquet, Juliette Pavie, Pierre Palmer, François Barbier, Stéphane Legriel, Julien Mayaux, Jean Michel Molina, Benoît Schlemmer, Elie Azoulay

**Affiliations:** 1 Service de réanimation médicale, AP-HP, Hôpital Saint-Louis, 1 avenue Claude Vellefaux, Université Paris-7 Paris-Diderot, UFR de Médecine, 75010 Paris, France; 2 Service de maladies infectieuses, AP-HP, Hôpital Saint-Louis, 1 avenue Claude Vellefaux, Université Paris-7 Paris-Diderot, UFR de Médecine, 75010 Paris, France; 3 Service de virologie, AP-HP, Hôpital Saint-Louis, 1 avenue Claude Vellefaux, Université Paris-7 Paris-Diderot, UFR de Médecine, 75010 Paris, France

## Abstract

**Introduction:**

The widespread use of highly active antiretroviral therapy (ART) has reduced HIV-related life-threatening infectious complications. Our objective was to assess whether highly active ART was associated with improved survival in critically ill HIV-infected patients.

**Methods:**

A retrospective study from 1996 to 2005 was performed in a medical intensive care unit (ICU) in a university hospital specialized in the management of immunocompromised patients. A total of 284 critically ill HIV-infected patients were included. Differences were sought across four time periods. Risk factors for death were identified by multivariable logistic regression.

**Results:**

Among the 233 (82%) patients with known HIV infection before ICU admission, 64% were on highly active ART. Annual admissions increased over time, with no differences in reasons for admission: proportions of patients with newly diagnosed HIV, previous opportunistic infection, CD4 counts, viral load, or acute disease severity. ICU and 90-day mortality rates decreased steadily: 25% and 37.5% in 1996 to 1997, 17.1% and 17.1% in 1998 to 2000, 13.2% and 13.2% in 2001 to 2003, and 8.6% in 2004 to 2005. Five factors were independently associated with increased ICU mortality: delayed ICU admission (odds ratio (OR), 3.04; 95% confidence interval (CI), 1.29 to 7.17), acute renal failure (OR, 4.21; 95% CI, 1.63 to 10.92), hepatic cirrhosis (OR, 3.78; 95% CI, 1.21 to 11.84), ICU admission for coma (OR, 2.73; 95% CI, 1.16 to 6.46), and severe sepsis (OR, 3.67; 95% CI, 1.53 to 8.80). Admission to the ICU in the most recent period was independently associated with increased survival: admission from 2001 to 2003 (OR, 0.28; 95% CI, 0.08 to 0.99), and between 2004 and 2005 (OR, 0.13; 95% CI, 0.03 to 0.53).

**Conclusions:**

ICU survival increased significantly in the highly active ART era, although disease severity remained unchanged. Co-morbidities and organ dysfunctions, but not HIV-related variables, were associated with death. Earlier ICU admission from the hospital ward might improve survival.

## Introduction

In industrialized countries, treatment advances have converted AIDS from a disease that was almost universally fatal within a few months to a chronic disease that can be controlled for many years [1]. A major turning point was the introduction of antiretroviral therapy (ART) in the mid-1990s. ART has increased the life expectancy of patients who are infected with the HIV and has reduced the incidence of life-threatening complications of AIDS [2-4]. In countries where ART is widely available, even patients with advanced immunosuppression may enjoy prolonged survival [5]. However, life-threatening infectious or toxic complications still arise frequently [6-8]. Nevertheless, both the prevalence of opportunistic infections and the mortality rates have fallen sharply since the early years of the HIV epidemic, and the proportion of HIV-infected patients who die from AIDS-defining illnesses has declined [9-11].

Intensive care unit (ICU) management of HIV-infected patients was widely perceived as futile in the 1980s, by both physicians and patients, as ICU mortality was about 70% [1,4]. Later on, increasing numbers of HIV-infected patients were admitted to the ICU, and survival rates improved over time in the late 1980s and early 1990s [12-14]. Subsequently, the major benefits of ART therapy prompted several groups to compare ICU admission patterns and survival in the pre-ART and post-ART eras. The results were conflicting, with some studies finding no significant differences [8,14] and another study showing a significant increase in survival (from 49 to 71%), perhaps associated with a sharp increase in ICU admissions for non-HIV-related diseases (from 12 to 67%) [12]. Now, however, the benefits of ART are well established, and the ART era is a decade long. An appraisal of changes in ICU admission patterns and survival over this ART era is therefore timely.

The objective of the present study was to compare ICU admission patterns, survival, and risk factors for ICU mortality in HIV-infected patients over four consecutive time periods spanning the decade from 1996 to 2005. During this decade, ART has been widely available to HIV-infected patients in France, where treatment costs are entirely covered by a universal health insurance system.

## Materials and methods

This retrospective observational cohort study was conducted in the ICU of the Saint-Louis Teaching Hospital in Paris, France. The ethics committee of the Bichat Hospital (CEERB) approved the study. All HIV-infected patients admitted to the ICU between 1996 and 2005 were included. In our hospital, as soon as the Department of Infectious Disease requests an HIV-infected patient's referral to the ICU, admission to the ICU is unrestrictedly and immediately scheduled.

The data reported in Tables 1 and 2 were abstracted from the medical records, as well as from the history of AIDS-defining illnesses. ART was defined as a combination of at least three antiretroviral drugs belonging to at least two classes (that is, nucleoside reverse transcriptase inhibitors, non-nucleoside reverse transcriptase inhibitors, or protease inhibitors). ART was considered effective if the CD4 cell count was no lower than 200 × 10^9 ^cells/l and/or the HIV load was no higher than 200 copies/ml. Direct admission to the ICU was defined as an admission to the ICU directly from the emergency department or the prehospital mobile medical team (SAMU). The nature and duration of life-supporting treatments used throughout the ICU stay were recorded. The cause of the critical illness was determined based on clinical, radiographic, microbiological, and cytologic findings, and then validated by a multidisciplinary panel according to predefined criteria. Daily discussions between intensivists, consultants in infectious diseases and adequate specialists lead to consensus about definite diagnoses that are mentioned in Table 1. Diagnoses of infectious diseases were performed as previously described [15]. Macrophage activation syndrome was diagnosed according to the 2004 hemophagocytic lymphohistiocytosis criteria in patients with cytopenia, fever, and picture of hemophagocytosis in a bone marrow or liver specimen [16].

**Table 1:** Characteristics of the 284 HIV-positive patients admitted to the ICU between 1996 and 2005

Variable	Survived the ICU (n = 245)	Died in the ICU (n = 39)	Odds ratio (95% confidence interval)	*P *value
Age	42.6 (36.4 to 48.6)	41.3(36.1 to 49)	1 (0.9 to 1)	0.9
Males	176 (71.8)	27(69.2)	0.9 (0.4 to 1.8)	0.74
Period of ICU admission				
1996 to 1997	18 (7.3)	6 (15.4)	Ref	-
1998 to 2000	63 (25.7)	13 (33.3)	0.62 (0.21 to 1.86)	0.39
2001 to 2003	79 (32.2)	12 (30.8)	0.46 (0.15 to 1.38)	0.16
2004 to 2005	85 (34.7)	8 (20.5)	0.28 (0.09 to 0.91)	0.03
Co-morbidities				
COPD	12 (4.9)	3 (7.7)	1.6 (0.4 to 6.1)	0.5
Hepatic cirrhosis	19 (7.8)	9 (23)	3.6 (1.5 to 8.6)	0.005
Chronic C hepatitis infection	45 (18.4)	11(28.2)	1.7 (0.8 to 3.8)	0.1
Chronic B hepatitis infection	41 (16.7)	9 (23.1)	1.5 (0.7 to 3.4)	0.3
Chronic renal failure	18 (7.3)	1 (2.6)	0.3 (0.04 to 2.6)	0.3
Kaposi sarcoma	23 (9.4)	9 (23.1)	2.9 (1.2 to 6.8)	0.01
Psychiatric disorders	77 (31.4)	8 (20.5)	0.56 (0.25 to 1.3)	0.2
Homeless	22 (9)	4 (10.3)	1.2 (0.4 to 3.6)	0.8
HIV-related characteristics				
Time since HIV diagnosis (months)	70.5 (4 to 146)	88 (13 to 149.5)	1 (0.9 to 1)	0.7
HIV diagnosis within past 60 days	51 (21.1)	5 (14.3)	0.6 (0.2 to 1.7)	0.3
CD4^+ ^cell count	96 (23.5 to 289)	65 (32 to 287)	0.9 (0.9 to 1)	0.5
CD4^+ ^cell count <200	162 (66.2)	27 (68.7)	1.12 (0.51 to 2.5)	0.77
Previous opportunistic infections	121 (49.4)	18 (46.1)	0.9 (0.4 to 1.7)	0.7
Viral load (× 1,000 log_10_/ml)	53.8 (0.5 to 252)	28.1 (0 to 10825)	1	0.2
On HAART at ICU admission^a^	125 (51)	25 (64.1)	1.7 (0.8 to 3.4)	0.13
Viral replication controlled	58 (46.4)	10 (43.5)	0.9 (0.4 to 2.2)	0.8
Cotrimoxazole prophylaxis	79 (32.2)	15 (38.4)	1.3 (0.6 to 2.6)	0.4
ICU admission				
Direct ICU admission	135 (55.1)	15 (38.5)	0.51 (0.25 to 1.02)	0.05
Hospital to ICU admission (days)	0 (0 to 2)	1 (0 to 8)	1.05/day (1.01 to 1.08)	0.01
Main reason for ICU admission				
Acute respiratory failure	145 (59.2)	22 (56.4)	0.9 (0.4 to 1.8)	0.7
Coma	71 (28.9)	20 (51.3)	2.6 (1.3 to 5.1)	0.006
Sepsis^b^	48 (19.6)	20 (51.3)	4.3 (2.1 to 8.7)	0.0001
Shock^c^	34 (13.9)	25 (64.1)	11.1 (5.2 to 23.4)	0.0001
Renal failure	32 (13.1)	15 (38.5)	4.2 (2 to 8.8)	0.0002
Metabolic abnormalities	19 (7.8)	13 (33.3)	5.9 (2.6 to 13.4)	0.0001
Liver failure	9 (3.7)	14 (35.9)	14.7 (5.8 to 37.3)	0.0001
Definite diagnoses				
Infection	103 (42)	26 (66.7)	2.8 (1.3 to 5.6)	0.005
Septic shock^d^	12 (4.9)	20 (51.3)	20.4 (8.7 to 48)	0.0001
Bacterial pneumonia	84 (34.3)	10 (25.6)	0.7 (0.3 to 1.4)	0.3
Pneumocystis pneumonia	50 (20.4)	3 (7.7)	0.3 (0.1 to 1.1)	0.07
Cerebral toxoplasmosis	15 (6.1)	2 (2.6)	0.4 (0.05 to 3.1)	0.4
Status epilepticus	19 (7.8)	3 (7.7)	1 (0.3 to 3.5)	0.9
Meningitis	18 (7.3)	2 (5.1)	0.7 (0.1 to 3.1)	0.6
Cerebral hemorrhage	4 (1.6)	5 (12.8)	8.9 (2.3 to 34.6)	0.002
Multiple organ failure	5 (2)	24 (61.5)	76.8 (25.7 to 229.7)	0.0001
Macrophage activation syndrome	10 (4)	6(15.4)	4.27 (1.5 to 12.5)	0.008
Life-supporting procedures				
Mechanical ventilation	86 (35.1)	38 (97.4)	70.2 (9.5 to 521)	0.0001
Renal replacement therapy	17 (6.4)	14 (35.9)	7.5 (3.3 to 17)	0.0001
Vasopressors	33 (13.5)	31 (79.5)	24.9 (10.5 to 59)	0.0001
Duration of life support (days)	0 (0 to 3)	3 (1 to 7.7)	1.07 (1.02 to 1.1)	0.002

Data presented as median (interquartile range) or number (percentage). ICU, intensive care unit; COPD, chronic obstructive pulmonary disease; HAART, highly active antiretroviral therapy. ^a^On HAART at ICU admission for >30 days. ^b^Defined as clinically or microbiologically documented infection with systemic inflammatory response syndrome. ^c^Defined as systolic arterial pressure <80 mmHg despite adequate fluid resuscitation. ^d^Defined as sepsis-induced hypotension persisting despite adequate fluid resuscitation.

**Table 2:** Changes over the four study periods

Variable	1996 to 1997 (n = 24, 8.5%)	1998 to 2000 (n = 76, 26.8%)	2001 to 2003 (n = 91, 32%)	2004 to 2005 (n = 93, 32.7%)	*P *value
Mean age (years)	35 (30 to 41)	40 (35 to 48)	43 (36 to 49)	44 (40 to 50)	0.0002
African ethnicity	6 (25)	11 (14.5)	26 (28.6)	34 (36.6)	0.01
Co-morbidities					
Hepatic cirrhosis	1 (4.2)	11 (14.5)	10 (11)	6 (6.4)	0.24
Chronic hepatitis C infection	2 (8.3)	19 (25)	23 (25.3)	12 (12.9)	0.03
Chronic hepatitis B infection	3 (12.5)	16 (21)	15 (16.5)	16 (17.2)	0.76
Homeless	0	4 (5.3)	8 (8.8)	14 (15)	0.05
HIV-related characteristics					
Time from diagnosis (months)	46 (2 to 125)	70 (3 to 147)	74 (6 to 135)	81 (6 to 170)	0.42
HAART administration^a^	9 (37.5)	40 (52.6)	52 (57.1)	49 (52.7)	0.39
New HIV diagnosis^b^	6 (25)	17 (22.4)	18 (20.2)	15 (17)	0.77
CD4^+ ^cell count >200/mm^3^	6 (26.1)	26 (35.1)	23 (27.7)	30 (36.1)	0.55
Direct admission to the ICU	16 (66.7)	31 (40.8)	40 (44)	44 (47.3)	0.15
Main reason for ICU admissions					
Sepsis	7 (29.2)	15 (19.7)	23 (25.3)	23 (24.7)	0.74
Bacterial pneumonia	7 (29.2)	25 (32.9)	32 (35.2)	30 (32.3)	0.94
Pneumocystis pneumonia	4 (16.7)	17 (22.4)	14 (15.4)	18 (19.3)	0.69
Cerebral toxoplasmosis	1 (4.2)	4 (5.3)	5 (5.5)	6 (6.4)	0.97
SAPS II	53 (33 to 60)	44 (31 to 57)	46 (32 to 55)	48 (35 to 61)	0.44
Life-supporting procedures					
Mechanical ventilation	14 (58.3)	38 (50)	40 (44)	32 (34.4)	0.08
Renal replacement therapy	3 (12.5)	8 (10.5)	13 (14.3)	7 (7.5)	0.52
Vasopressors	8 (33.3)	18 (23.7)	21 (23)	17 (18.3)	0.46
ICU mortality	6 (25)	13 (17.1)	12 (13.2)	8 (8.6)	0.01

Data presented as median (interquartile range) or number (percentage). HAART, highly active antiretroviral therapy; ICU, intensive care unit; SAPS II, Simplified Acute Physiological Score version II. ^a^HAART administration prescribed for more than 30 days before ICU admission. ^b^Diagnosis of HIV infection within past 60 days.

Vital status at ICU discharge and then 3 and 12 months later was available for all patients. ICU mortality was our main outcome variable of interest.

### Statistical analysis

Results are reported as the median (interquartile range (IQR)) or as the number (percentage). Patient characteristics were compared using the chi-square test or Fisher's exact test, as appropriate, for categorical variables and using the nonparametric Wilcoxon's rank sum test or the Kruskal-Wallis test for continuous variables.

To investigate associations between patient characteristics and ICU death, we first performed bivariate analyses to look for a significant influence of each variable on ICU mortality by logistic regression, as measured by the estimated odds ratio (OR) with the 95% confidence interval (CI). Variables yielding *P *values no greater than 0.20 in the bivariate analyses were entered into a multiple logistic regression model (backward procedure) in which ICU mortality was the primary outcome. Entered variables were dropped if they were no longer significant when other variables were added. Variables entered into the final model are presented in Table 3. The variable ART was forced into the multivariable analysis.

**Table 3:** Multivariable analysis to identify factors independently associated with ICU death

	Odds ratio	95% confidence interval	*P *value
Associated with survival			
ICU admission 1996 to 1997	-	Reference	Reference
ICU admission 1998 to 2000	0.32	0.09 to 1.15	0.08
ICU admission 2001 to 2003	0.28	0.08 to 0.99	0.004
ICU admission 2004 to 2005	0.13	0.03 to 0.53	0.005
Associated with death			
Hepatic cirrhosis	3.78	1.21 to 11.84	0.02
Delayed ICU admission	3.04	1.29 to 7.17	0.01
ICU admission for coma	2.73	1.16 to 6.46	0.02
Acute renal failure	4.21	1.63 to 10.92	0.003
Severe sepsis	3.67	1.53 to 8.80	0.004
Anti-retroviral therapy	1.60	0.60 to 4.29	0.36

The multivariable model did not include seven patients because of missing data. Goodness of fit chi-square (Hosmer-Lemeshow statistics)*P *= 0.23. ICU, intensive care unit.

Finally, we estimated probabilities of survival according to the Kaplan-Meier method with log-rank tests. In patients with multiple ICU stays, only the first ICU stay was included. All tests were two-sided, and *P *< 0.05 was considered statistically significant. Analyses were carried out using the SAS 9.1 software package (SAS Institute, Cary, NC, USA).

## Results

Over the 10-year study period, 284 HIV-infected patients were admitted to our ICU for life-threatening events. As shown in Table 1, the most common co-morbidities included hepatitis C (19.7%), hepatitis B (17.6%), and psychiatric disorders (29.9%). The median time from the diagnosis of HIV infection to ICU admission was 74 months (IQR, 4.7 to 147 months). In 56 (19.7%) patients, the diagnosis of HIV infection was made within 60 days before ICU admission. About one-half of the patients (n = 150) were on ART at ICU admission, including 68 who had viral load and/or CD4 count values indicating disease control. The median CD4 count was 92/mm^3 ^(IQR, 27 to 289/mm^3^). ART was not interrupted in the ICU unless drug-related toxicity occurred. In case ART could have worsened an acute organ dysfunction, the drug was either withdrawn or changed to another from the same class.

As reported in Table 1, acute respiratory failure was the main reason for ICU admission (58.8%), followed by neurological disease (32%) and sepsis (23.9%). Bacterial pneumonia was the most common infectious event, and status epilepticus the most common non-infectious event. In patients with documented bacterial infections, *Streptococcus pneumoniae *(n = 41), *Escherichia coli *(n = 14), and *Pseudomonas aeruginosa *(n = 11) predominated. *Toxoplasma gondii *(n = 17) was the most frequent documented parasite and *pneumocystis jirovecii *(n = 53) was the most frequent fungal agent, followed by *Cryptococcus neoformans *(n = 8). In the most recent period, more patients were admitted for a non-AIDS-related event. Opportunistic infections occurred only in patients who had discontinued ART or had not been diagnosed with AIDS before ICU admission. Only seven admissions were related to ART-related toxicity.

**Figure 1:**
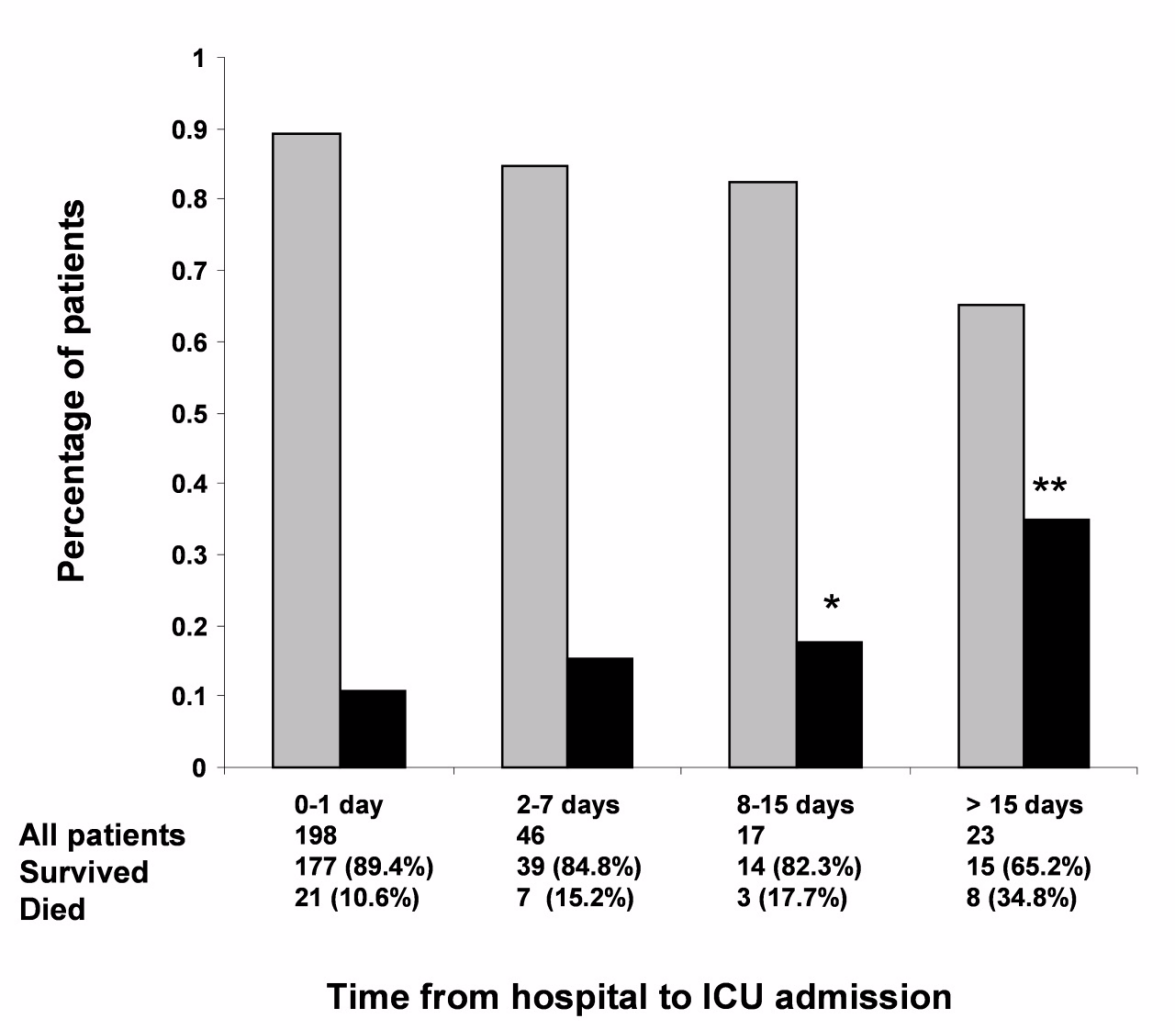
**Intensive care unit mortality and time from hospital to intensive care unit admission**. Relationship between intensive care unit (ICU) mortality and time from hospital to ICU admission. Gray bars, patients who survived; black bars, patients who died. *P < 0.05, **P < 0.01.

The average annual number of ICU admissions of HIV-infected patients increased from one study period to the next (Table 2). In parallel, ICU mortality decreased from the earliest to the latest periods (25% for 1996 to 1997, 17.1% for 1998 to 2000, 13.2% for 2001 to 2003 and 8.6% for 2004 to 2005, respectively). Figure 1 shows the relationship between ICU mortality and the time from hospital to ICU admission. Among HIV-related variables, ART was more common in the most recent period; however, no significant changes occurred over time from diagnosis, CD4 cell count, HIV load, and number of patients admitted for their first AIDS-defining episode.

Mechanical ventilation was needed in 124 (43.6%) patients, vasopressors in 64 (22.5%) patients, and renal replacement therapy in 31 (10.9%) patients. The median duration of supportive care was 1 day (IQR, 0 to 3 days). The use of mechanical ventilation, vasopressors, and renal replacement therapy remained unchanged from one time period to the next. The overall median Simplified Acute Physiological Score version II was 49 (IQR, 31 to 54), with no significant changes over time. The median ICU stay length was 4 days (IQR, 2 to 7 days). The ICU and 90-day mortalities were 13.7% (39 deaths) and 14.8% (42 deaths), respectively.

**Figure 2:**
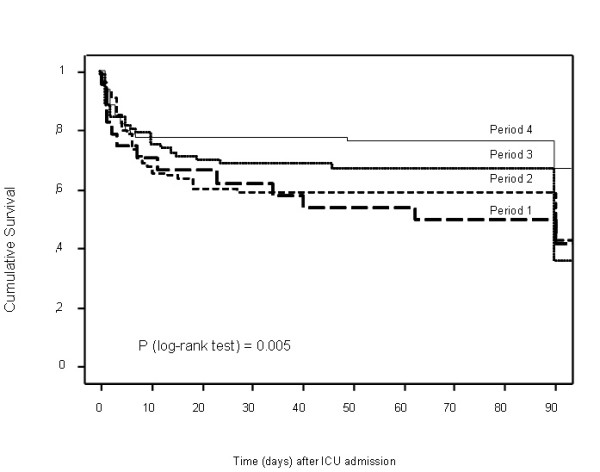
**Mortality according to period of intensive care unit admission**. The four study periods were: Period 1, 1996 and 1997 (solid line); Period 2, 1998 to 2001 (dotted line); Period 3, 2001 to 2003 (short dashes); and Period 4, 2004 and 2005 (long dashes).

By univariate analysis, Kaposi sarcoma was the only HIV-related factor associated with ICU mortality (Table 1). Mortality was higher in patients with cirrhosis, in those whose ICU admission occurred after a longer stay in the wards (Table 1), and in those with larger numbers of life-supporting treatments and longer times on life-supporting treatments. Mortality was significantly lower in patients admitted during the most recent period, compared with patients admitted in earlier periods (Figure 2).

Table 3 reports the results of the multivariable analysis. Five factors were independently associated with increased ICU mortality: delayed ICU admission (OR, 3.04; 95% CI, 1.29 to 7.17), acute renal failure (OR, 4.21; 95% CI, 1.63 to 10.92), hepatic cirrhosis (OR, 3.78; 95% CI, 1.21 to 11.84), ICU admission for coma (OR, 2.73; 95% CI, 1.16 to 6.46), and severe sepsis (OR, 3.67; 95% CI, 1.53 to 8.80). Admission to the ICU in the most recent period was independently associated with increased survival: admission from 2001 to 2003 (OR, 0.28; 95% CI, 0.08 to 0.99), and between 2004 and 2005 (OR, 0.13; 95% CI, 0.03 to 0.53).

## Discussion

Our study of characteristics and outcomes of 284 HIV-infected patients admitted to the ICU during the first 10 years of the ART era shows that HIV characteristics remained unchanged over time. Also, demographic and HIV characteristics were unrelated to survival. Neither did the severity of the acute illness requiring ICU admission change over time. Nevertheless, mortality decreased steadily, and the difference between the most recent period and earlier periods was statistically significant. Most deaths were ascribable to co-morbidities, organ dysfunctions, and delayed ICU admission.

The finding that HIV-related characteristics are not associated with mortality is a striking one since this has direct implication for patient management and triage. This could be ascribable to a better ICU management, with increased ability to perform infectious or non-infectious diagnoses in severely immunocompromised patients [1,4,17]. This observation is in agreement with the results of the ICU in other immunocompromised patients such as bone marrow transplant recipients or other patients with hematological malignancies [18-21].

Chronic active hepatitis and cirrhosis are emerging as challenging targets for improving the survival of HIV-infected patients. Co-infection with hepatitis viruses has been reported in about 20% of HIV-positive patients and is associated with decreased long-term survival rates [22]. Recent advances in targeted treatments for hepatitis B and hepatitis C may improve survival in the near future [23].

The decrease in mortality over time evidenced by our study cannot be ascribed to changes in patient selection for ICU admission, as no significant changes occurred in the burden of co-morbidities or in the HIV load and CD4 cell count. The increased number of ICU admissions of HIV-positive patients over time despite the stable incidence of HIV infection in our area suggests a longer survival of HIV-positive patients [14]. Our results agree with those of other studies showing better survival of critically ill HIV-positive patients [17]. Among critically ill patients who have co-morbidities, those with HIV infection may be more likely to survive than those who have chronic obstructive pulmonary disease, heart failure, or cancer [24-27].

ICU management was not different across the four study periods. The number of patients who received life-supporting treatments and the durations of those treatments remained unchanged over time. ICU admission occurred earlier in the more recent periods, however, and earlier admission was independently associated with better survival. Neither the reasons for ICU admission nor the nature of the acute events changed over time. In all four study periods, opportunistic infections occurred only in patients who had discontinued ART or did not have a diagnosis of AIDS before ICU admission [28]. Bacterial infections and non-infectious diseases were the main reasons for ICU admission in the other patients. The extraordinarily strong association between macrophage activation syndrome and death suggests a high risk of life-threatening malignancies among patients surviving HIV infection [16].

Our study has several limitations. First, the design was retrospective. All patients were managed at the same center using standardized written protocols, however, and no data were missing.

Second, increased survival could have been ascribable to differences in triage to ICU admission, as previously reported [29]. Three findings may not argue in favor of selection for ICU admission, however: the number of admitted patients increases over time; the time since HIV diagnosis, CD4 cell rate, viral load and opportunistic infections were not different across the four time periods, indicating that we probably have not selected patients based on HIV data; and our incentive to admit patients earlier clearly shows that, rather than denying ICU admission, we may be in favor of opening the ICU doors to HIV patients.

Third, ART use at ICU admission was not associated with ICU mortality. Most of our patients, however, were admitted for bacterial infections or non-HIV-related diseases. Moreover, our finding that opportunistic infections occurred only in patients who were not receiving ART offers hope for improving outcomes in HIV-positive patients via earlier detection of HIV infection.

Fourth, earlier ICU admission was associated with better survival but not with decreases in the use of mechanical ventilation, renal replacement therapy, and vasopressors [30]. Significant reductions in the need for medical ward admission of HIV-infected patients after the advent of ART have been reported [1]. ART has decreased the risk of immune suppression and AIDS development, diminished the incidence of opportunistic infections, and improved survival [13]. Despite the immunologic and virologic advantages conferred by ART, several recent studies find no improvement in hospital or short-term survival between patients receiving ART or not receiving ART at time of ICU admission [12,17].

Last, even though cardiovascular disease is emerging as a cause of morbidity and mortality in HIV-positive patients [31], none of our patients had cardiovascular disease as the reason for ICU admission, since patients with cardiovascular disease were admitted to a nearby hospital equipped with a cardiovascular unit.

## Conclusions

In summary, the past decade has witnessed both a steady increase in admissions of ICU-positive patients and a significant increase in their survival rates. Opportunistic infections occurred only in patients who were not receiving highly active ART. Patients on ART required ICU admission for bacterial infections or non-AIDS-related events. Our study suggests that earlier ICU admission of HIV-infected patients may improve survival. Raising awareness among emergency room physicians and emergency mobile-unit physicians that HIV-infected patients are now good candidates for ICU admission might help to achieve earlier admission. These hypotheses should be tested prospectively.

## Key messages

• Throughout the 10-year study period, annual admissions increased over time, with no differences in reasons for admission or proportions of patients with newly diagnosed HIV.

• ICU and 90-day mortality rates decreased steadily over the past decade (from 37.5 to 8.6%), with admission to the ICU in the most recent period being independently associated with increased survival.

• Delayed ICU admission was associated with increased ICU mortality.

## Abbreviations

AIDS: acquired immunodeficiency syndrome; ART: antiretroviral therapy; CI: confidence interval; HIV: human immunodeficiency virus; ICU: intensive care unit; IQR: interquartile range; OR: odds ratio.

## Competing interests

The authors declare that they have no competing interests.

## Authors' contributions

EA, JMM and BS contributed to study design and mentoring. IC, PP and JP contributed to data collection, interpretation and manuscript preparation. FB contributed to study design, data collection and preparation of the manuscript. JM and SL contributed to preparation of the manuscript, audit of the database, and statistical work. All authors contributed substantially to the submitted work and read and approved the final manuscript.
